# Ground state anomalies in SmB_6_

**DOI:** 10.1038/s41598-020-58172-7

**Published:** 2020-01-27

**Authors:** Anup Pradhan Sakhya, Kalobaran Maiti

**Affiliations:** Department of Condensed Matter Physics and Materials Science, Tata Institute of Fundamental Research, Homi Bhabha Road, Colaba, Mumbai, 400 005 India

**Keywords:** Condensed-matter physics, Theory and computation, Electronic properties and materials, Magnetic properties and materials, Topological matter

## Abstract

SmB_6_ has drawn much attention in recent times due to the discovery of anomalies in its ground state properties as well as prediction of topologically protected gapless surface states. Varied theories have been proposed to capture the ground state anomalies. Here, we studied the electronic structure of SmB_6_ employing density functional theory using different exchange correlation potentials, spin-orbit coupling and electron correlation strength. We discover that a suitable choice of interaction parameters such as spin-orbit coupling, electron correlation strength and exchange interaction within the generalized gradient approximation provides a good description of the spectral functions observed in the angle-resolved photoemission spectroscopy (ARPES) studies. The Fermi surface plots exhibit electron pockets around *X*-point and hole pockets around Γ*X* line having dominant Sm 4*f* character. These observations corroborate well with the recent experimental results involving quantum oscillation measurements, ARPES, etc. In addition to primarily Sm 4*f* contributions observed at the Fermi level, the results exhibit significantly large contribution from B 2*p* states compared to weak Sm 5*d* contributions. This suggests important role of B 2*p* - Sm 4*f* hybridization in the exotic physics of this system.

## Introduction

Mixed valent Kondo insulators have attracted tremendous attention followed by the discovery of varied exotic ground state properties arising from strong Coulomb repulsion among 4*f* electrons, often termed as ’electron correlation’ and the hybridization of 4*f* states with the conduction electronic states^1–5^. SmB_6_ is one such material^1,3,6^, exhibits metallicity at room temperature and becomes insulating below 40 K, which is believed to arise due to the formation of many body singlet states constituted by localized Sm 4*f* electrons and the dispersive Sm 5*d* electrons. Various experimental studies, however, revealed anomalies at low temperatures such as finite linear specific heat coefficient, bulk optical conductivity below the expected charge gap, quantum oscillations within the insulating phase, and saturation of resistance below 4 K, which was attributed to the presence of *in-gap* states within the charge gap^7^.

First principles calculations of the electronic structure of SmB_6_ predicted non-trivial *Z*_2_ topology that may host topologically protected metallic surface states leading to a saturation of resistance at low temperatures^8,9^. Subsequent transport^10–12^ and angle-resolved photoemission spectroscopy (ARPES)^13–16^ measurements on SmB_6_ supports the presence of topologically ordered metallic surface states. Employing spin-resolved ARPES measurements, Xu *et al*.^17^ showed that the metallic surface states in this material are spin polarized and the spin texture fulfills the condition that the surface states are protected by time-reversal symmetry suggesting SmB_6_ to be an example of a topological Kondo insulator.

Quantum oscillation experiments by Li *et al*. exhibit signature of two dimensional Fermi surfaces on (100) and (101) surface planes supporting the presence of topological surface states within the bulk hybridization gap^18^. Recent studies, however, argued that the observed metallic surface states have trivial origin rendering SmB_6_ a trivial surface conductor^19^. Torque magnetometry experiments by Tan *et al*.^20^ exhibit angular dependence of de Haas van Alphen oscillations suggesting three dimensional nature of the observed Fermi surfaces. It was suggested that the high frequency quantum oscillations originate from three dimensional Fermi surface resembling the *d* type conduction electron Fermi surface in metallic LaB_6_^20,21^. In order to explain low temperature anomalies, some groups proposed Fermi surfaces due to neutral fermionic composite exciton^22,23^. Recently, Harrison *et al*.^24^ has shown the presence of highly asymmetric nodal semi-metal phase existing over certain region of momentum space in bulk SmB_6_, where the node is pinned to the un-hybridized *f*-level, casting doubt over the necessity of a neutral Fermi surface.

Evidently, the puzzles in SmB_6_ are far from resolved. We have calculated the electronic structure of SmB_6_ employing density functional theory (DFT) using various exchange correlation potentials. We discover that the ground state electronic structure of SmB_6_ for a suitable choice of electronic interaction parameters provides a remarkable description of the experimental results.

**Figure 1:**
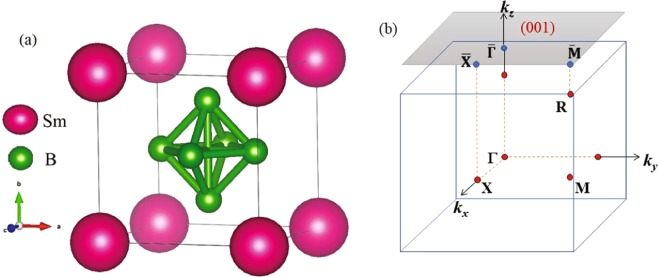
(**a**) Crystal structure of SmB_6_ is shown exhibiting simple cubic symmetry. (**b**) The bulk and surface Brillouin zones are shown along with various high symmetry points.

SmB_6_ forms in cubic structure with Sm atoms at the corner of the cube and B_6_ octahedra at the body center as shown in Fig. 1a. The crystal structure possesses inversion symmetry. We have used experimentally observed lattice constants of SmB_6_^25^ for our calculations. In Fig. 1b, we show the bulk Brillouin zone (BZ) having cubic symmetry; the centre of the BZ is the Γ point and the edge centres, face centers & corners are denoted by *M*, *X* & *R* points, respectively. The shaded area on top represents the projection of the bulk BZ at the surface. The high symmetry points on the surface BZ are represented by $\bar{\Gamma }$, $\bar{X}$ and $\bar{M}$. The energy bands were calculated along various *k*-vectors shown in the figure.

## Results and Discussions

**Figure 2:**
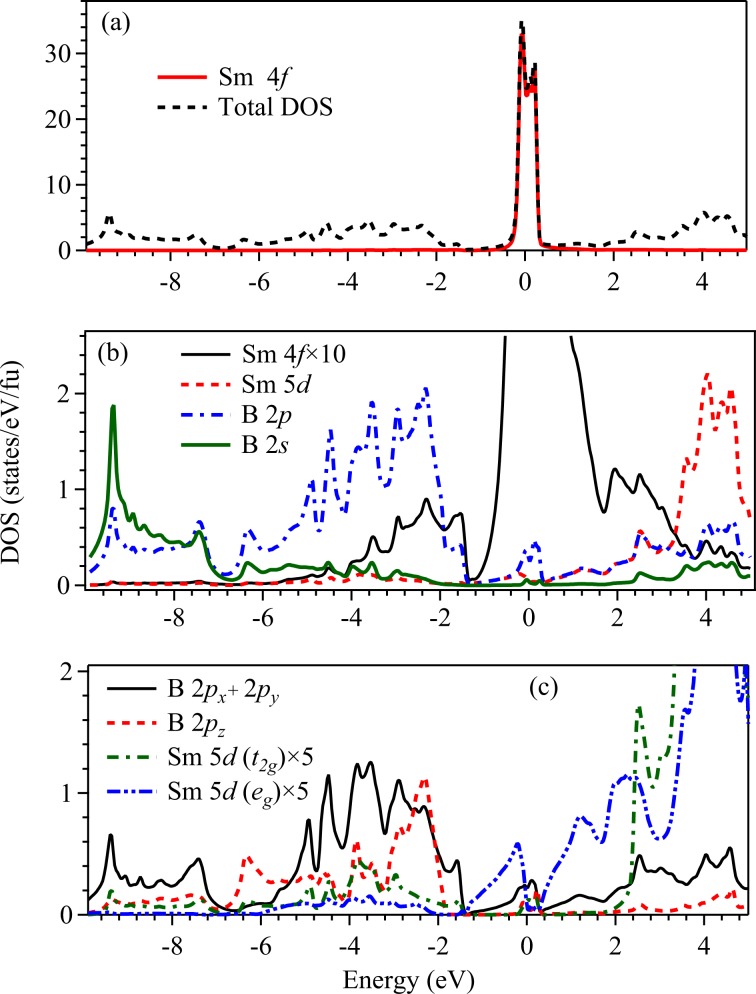
Calculated (**a**) total density of states (Total DOS) are shown by dashed line and Sm 4*f* partial density states (PDOS) are shown by solid line. (**b**) Solid line represents the PDOS of Sm 4*f* after rescaling it by 10 times for better comparison. PDOS of Sm 5*d* (dashed line), B 2*p* (dot-dashed line) and B 2*s* (green solid line) are also shown. Significant hybridization of B 2*p* and Sm 4*f* is evident. (**c**) B (2*p*_*x*_ + 2*p*_*y*_) PDOS (solid line), B 2*p*_*z*_ PDOS (dashed line), and 5 times of Sm 5*d* PDOS with *t*_2*g*_ (dot-dashed line) and *e*_*g*_ (dot-dot-dashed line) symmetries.

In Fig. 2a, we show the calculated total density of states (Total DOS) obtained using generalized gradient approximation (GGA) for the exchange correlation potential. Partial density of states (PDOS) have been calculated by projecting the eigenstates onto the atomic orbitals. The calculations converged to a metallic ground state with huge intensity at the Fermi level, *E*_*F*_ as evident in the figure. The region near *E*_*F*_ (−0.3 eV to 0.4 eV) is contributed mostly by Sm 4*f* states and the lower energy part of the valence band (VB) region (below −1 eV) is dominated by the contributions from B 2*p* states with some contribution from B 2*s* states as shown in Fig. 2b,c. The B 2*s* PDOS predominantly appear in the energy range from −7 eV to −10 eV.

In Fig. 2b, we show Sm 5*d*, B 2*p* contributions along with rescaled Sm 4*f* PDOS to compare the energy distribution of the density of states. It is evident that the energy distribution of the Sm 4*f* contributions look similar to B 2*p* contributions, which is a signature of hybridization between Sm 4*f* - B 2*p* states. The bonding bands appear between −7 eV to −1.6 eV with a peak at about −2.5 eV and the anti-bonding bands appear above −1.6 eV. Sm 5*d* states are essentially unoccupied and contribute beyond 3 eV above *E*_*F*_. There are weak Sm 5*d* contributions within the valence band regime suggesting finite coupling of Sm 5*d* − B 2*p* states. Interestingly, the Sm 5*d* PDOS observed here look very similar to the *d* PDOS in other hexaborides such as LaB_6_, CaB_6_^26–28^ where 4*f* contributions are not present. This suggests that *d*-*p* hybridizations are quite similar in this class of materials and 4*f* states may not be influencing the *d* states significantly. The energy distribution of various PDOS also manifests similar scenario.

In the crystal structure of SmB_6_, Sm ions at each corner is surrounded by eight B_6_ clusters located at the center of the cube and hence, Sm sites will experience cubic crystal field. Sm 5*d* orbitals have large radial extensions and hence, will experience strong crystal field effect. The cubic crystal field splits the Sm 5*d* levels into a doubly degenerate *e*_*g*_ band and a triply degenerate *t*_2*g*_ band. For the axis system similar to the crystal lattice axis and boron clusters located at the center of the cube, *e*_*g*_ states will have orbital lobes away from the clusters and hence, the energy for *e*_*g*_ electrons will be lower (weaker Coulomb repulsion energy) than the *t*_2*g*_ electrons possessing orbital lobes along the anion clusters due to the repulsion of the *d* electrons and negative ligand charges^29^. Thus, Sm 5*d* (*e*_*g*_) states are partially occupied and contribute at *E*_*F*_. Sm 5*d* (*t*_2*g*_) bands are essentially unoccupied lying high in energy (above 2.4 eV) in the conduction band as shown in Fig. 2c. B (2*p*_*x*_ + 2*p*_*y*_) PDOS shown in Fig. 2c exhibit similar energy distribution of Sm 4*f* PDOS indicating stronger hybridization with these states. B 2*p*_*z*_ seem to have significant hybridization with the Sm 5*d* states. From the PDOS plots, we find that the major contribution to the total DOS at *E*_*F*_ [*N*(*E*_*F*_)] comes from the Sm 4*f* states (98.6%), while the contribution from the Sm 5*d* and B 2*p* states are 0.16% and 1.1%, respectively. The contribution from B (2*p*_*x*_ + 2*p*_*y*_) is more near *E*_*F*_ than B 2*p*_*z*_ states.

**Figure 3:**
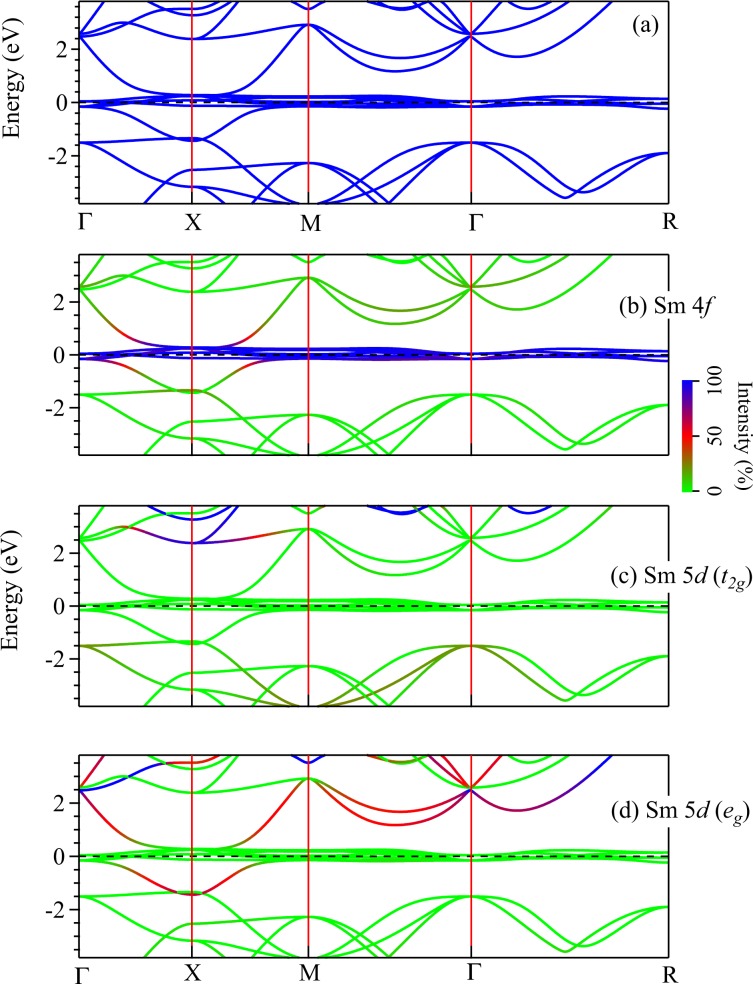
(**a**) Energy band structure derived using GGA method along various *k*-vectors. Contribution of (**b**) Sm 4*f*, (**c**) Sm 5*d* (*t*_2*g*_), and (**d**) Sm 5*d* (*e*_*g*_) states to various energy bands are also shown [Sm 5*d* contributions are rescaled by a factor of 2 for clarity].

The calculated energy band structure along high symmetry directions are shown in Fig. 3. While the DOS provide information about the contribution of various electronic states as a function of energy (*k*-integrated results), the band structure provides the *k*-resolved information that can be compared directly with the data from angle resolved photoemission spectroscopy (ARPES) measurements. In order to get more information regarding the contribution from different states, we have plotted the band structure weighted by the band-characters. The energy bands near *E*_*F*_ exhibit minimal dispersion indicating high degree of local character of the corresponding electronic states. From the colour plots in Fig. 3b, it is clear that these valence states possess essentially Sm 4*f* character as also manifested in the PDOS plots shown in Fig. 2.

A highly dispersive energy band crosses the Fermi level along Γ*X* and *XM* directions, and hybridizes with the Sm 4*f* bands. From the symmetry analysis shown in Fig. 3c,d, we observe that the bands having *e*_*g*_ symmetry hybridizes with Sm 4*f* bands; *t*_2*g*_ bands appear at higher energies. The projected band characters provide signature of band inversion near the Fermi level. In addition, at about −1.5 eV, two bands touch each other at *X*-point. Transport^7,30,31^ and optical measurements^32–34^ suggest insulating behavior at temperatures below 50 K, which is different from the DFT results presented so far. Moreover, Sm is a heavy element (*Z* = 62) and hence, spin orbit coupling (SOC) is expected to play an important role in the electronic properties of this material (Δ ∝ *Z*^4^). As a first step, we calculated the electronic structure including SOC and different exchange correlation potentials.

**Figure 4:**
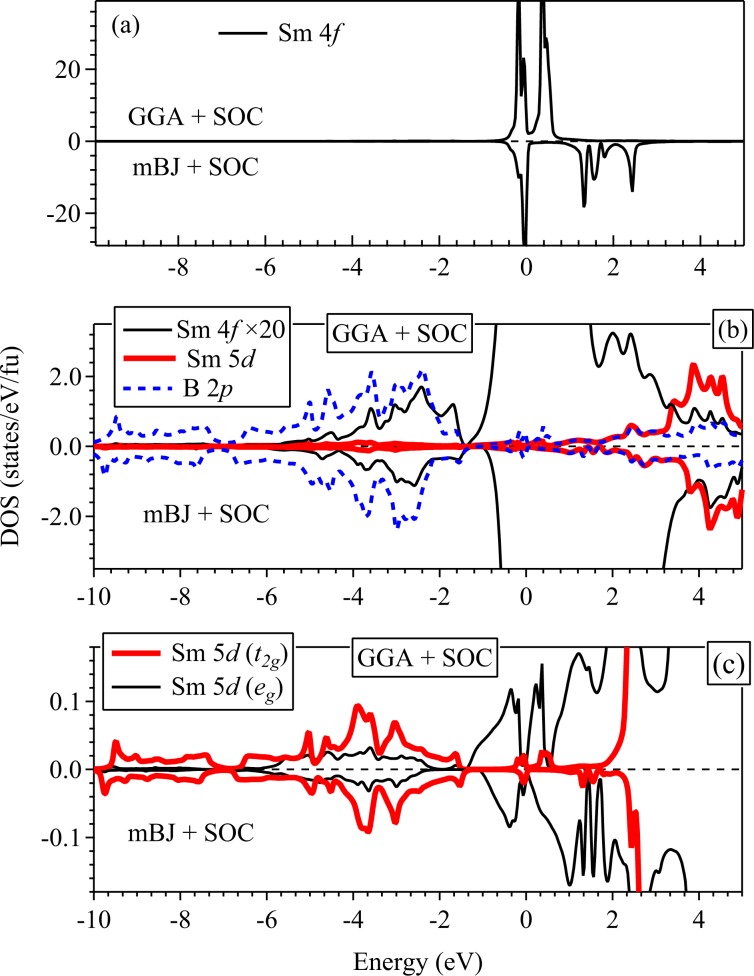
Calculated partial density of states of (**a**) Sm 4*f*, (**b**) Sm 4*f* (rescaled by 20 times shown by thin black solid lines), Sm 5*d* (thick red solid lines), B 2*p* (blue dashed lines), and (**c**) Sm 5*d* (*t*_2*g*_) (thick red solid lines) and Sm 5*d* (*e*_*g*_) (thin black solid lines) states. GGA + SOC results are shown along positive *y*-axis and mBJ + SOC results are shown along the negative axis for the sake of comparison.

The DOS and PDOS calculated with the inclusion of spin-orbit coupling is shown in Fig. 4 exhibiting significant changes near *E*_*F*_. The Sm 4*f* bands split into Sm 4*f*_5∕2_ (*J* = 5/2) and Sm 4*f*_7∕2_ (*J* = 7/2) levels as shown in Fig. 4a. The Sm 4*f*_5∕2_ band is partially occupied and lie in the energy range −0.4 eV to 0.1 eV, while Sm 4*f*_7∕2_ band is empty and lie in the energy range 0.2 eV to 0.7 eV. In addition, there is a marginal decrease in Sm 4*f* contribution at *E*_*F*_ (from 98.6% to 97.5%) with consequent increase in the Sm 5*d* and B 2*p* contributions to 0.3% and 2%, respectively. The DOS at *E*_*F*_ exhibit a pseudogap like feature (peak-dip-peak structure).

In order to verify the effect of the approximations in exchange correlation potentials in the electronic structure, we calculated the electronic structure using modified Becke Johnson potential including SOC (mBJ + SOC) method; the results are shown in Fig. 4 with reverse axis direction for better comparison. The DOS near *E*_*F*_ appears similar in both the cases and the pseudogap like feature observed in GGA + SOC results survives along with a higher degree of particle-hole asymmetry. The Sm 4*f*, 5*d* and B 2*p* PDOS below *E*_*F*_ exhibit signature of strong covalency between Sm 5*d*-B 2*p* and Sm 4*f*-B 2*p* states as evident in Fig. 4b. Sm 5*d* states with *t*_2*g*_ and *e*_*g*_ symmetry are shown in Fig. 4c exhibiting sensitivity to the consideration of exchange correlation potential. *N*(*E*_*F*_) in mBJ + SOC results is composed primarily of Sm 4*f* states contributing 98.7%, with the second most prominent contribution from B 2*p* states (1%), while the contribution from Sm 5*d* states is only 0.1%.

**Figure 5:**
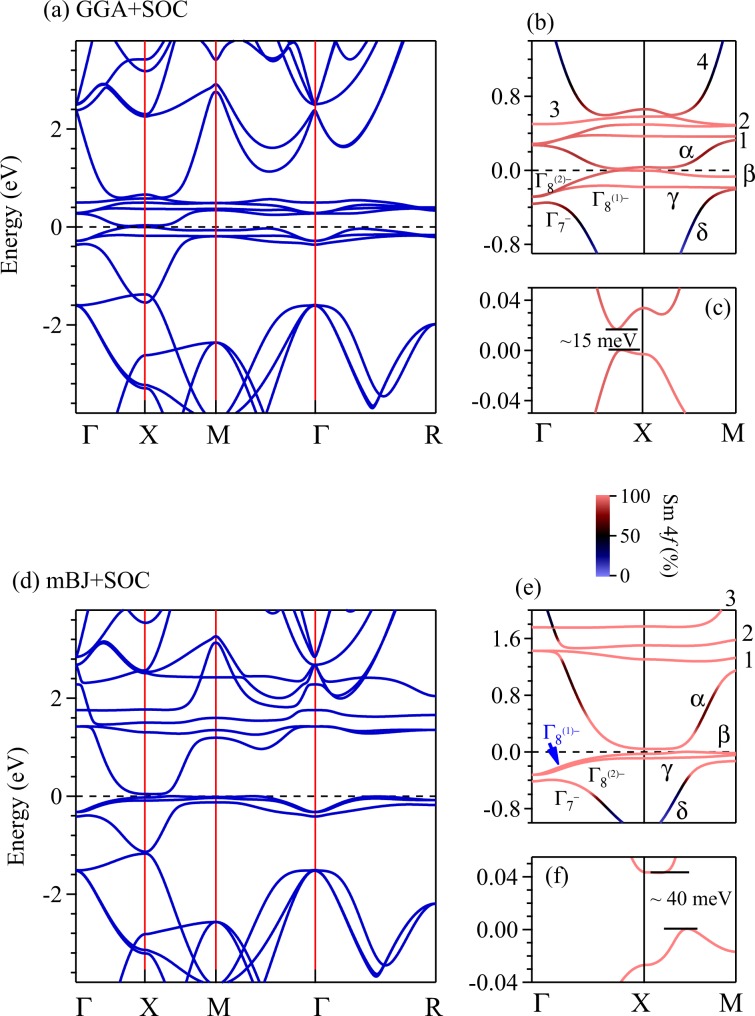
Energy band structure of SmB_6_ derived using GGA + SOC method are shown in (**a**–**c**). The results considering mBJ + SOC method are shown in (**d**–**f**). The energy region close to the Fermi level along Γ*XM* direction is shown in (**b**) and (**e**). Band gaps are shown in (**c**) and (**f**). Color scale provide relative contribution of various electronic states to the energy bands. Sm 4*f*_7∕2_ bands are denoted by 1, 2, 3 and 4. Sm 4*f*_5∕2_ bands are denoted by *α*, *β* and *γ*. *δ* represents Sm 5*d* band.

The band structure along with the band character plots are shown in Fig. 5. The inclusion of SOC leads to a splitting of the Sm 4*f* bands by about 0.6 eV in the GGA + SOC calculations shown in Fig. 5a. In addition, a direct band gap of about 15 meV opens up along Γ − *X* direction (see Fig. 5c) consistent with earlier results^35^. Based on ARPES measurements, Frantzeskakis *et al*.^16^ proposed that the states at the *X* point are essentially bulk Sm 5*d* states and the Fermi level lies at about 20 meV below the top of the valence band. The estimated band gap from their experimental results found to be within about 5–10 meV along *XM* and Γ−*X* directions; our theoretical results appears remarkably consistent with these experimental observations. We find that the bands exhibiting the gap possess primarily Sm 4*f* character. Sm 4*f* bands cross *E*_*F*_ along the Γ − *X* direction forming tiny hole pockets and along *XM* line, there is an energy gap.

On the other hand, mBJ + SOC results exhibit Fermi level crossing along the *XM* line; the band gap in this case becomes indirect with an energy gap of about 40 meV, which is much larger than the ARPES results. There exists a tiny hole pocket around *XM* line with no Fermi surface along Γ*X* direction. The spin-orbit coupling leads to splitting of Sm 4*f* bands to 4*f*_5∕2_ & 4*f*_7∕2_ bands with a large SOC splitting of about 1.6 eV. Moreover, the multiplet splitting of 4*f*_7∕2_ bands (band numbers 1, 2, 3 and 4 in the figure) in mBJ + SOC bands is significantly higher than GGA + SOC results.

In both the cases (GGA & mBJ), the band structure near the Fermi energy is dominated by Sm 4*f*_5∕2_ states as also found in LDA + Gutzwiller results^35^. Both GGA + SOC and mBJ + SOC results exhibit signature of band inversion along the high symmetry directions, Γ*X* and *XM* (see Fig. 5b,e). This band inversion has been discussed by many authors and is a prerequisite for SmB_6_ to be a topological insulator. In Fig. 5b,e, we have denoted the bands as *α*, *β*, *γ*, *δ*, 1, 2, 3 and 4. In the vicinity of *X* point, *α*, *β* and *γ* bands have *f* orbital character, and the band *δ* has *d* orbital character. The conduction bands denoted by 1, 2, 3 and 4 correspond to the spin-orbit split 4*f*_7∕2_ states. While in GGA + SOC results, these four bands lie very close to *E*_*F*_, they are shifted above 1.4 eV in the mBJ + SOC results (the band 4 is shifted to 2.3 eV above *E*_*F*_ and not shown in the figure). Most of the 4*f* bands in mBJ + SOC results are not displaying significant dispersion implying strong atomic nature of Sm 4*f* electrons similar to the LDA + Gutzwiller results^35–37^.

In the GGA + SOC and mBJ + SOC data shown above, although the band inversion takes place between *α* band having negative parity and *δ* band having positive parity, the band gap appears between *α* and *β* bands, i.e., the two 4*f* bands having the same parity. Thus, the band inversion in SmB_6_ is complex and different from the band inversion observed in the typical topological insulators such as Bi_2_Se_3_ and Bi_2_Te_3_, where electron correlation is not important^38,39^. This makes SmB_6_ special having exoticity due to interplay between electron correlation and topological order^40^.

Sm 4*f* states experience stronger spin-orbit coupling than the crystal field effect due to their small orbital extension and screening of the crystal field by 5*d* electrons. Thus, 4*f* levels split into 4*f*_5∕2_ and 4*f*_7∕2_ bands with large energy separation. The crystal field splits Sm 4*f*_5∕2_ bands into a Γ_7_ doublet and a Γ_8_ quartet. Away from the Γ point, the Γ_8_ quartet further splits into ${\Gamma }_{8}^{1}$ and ${\Gamma }_{8}^{2}$ doublets, which is shown in the Fig. 5b,e, respectively^29^. SmB_6_ possesses inversion symmetry. *Z*_2_ topological invariants have been computed via parity analysis and found to be *Z*_2_ = 1^41–43^. Thus, SmB_6_ has been predicted to be a topologically non-trivial system with an odd number of gapless surface states.

While all the above conclusions are interesting and exhibit some features of the experimental observations, the experimental ARPES data^14,44,45^ are significantly different from the results discussed so far. The Sm 4*f* feature observed at around −1 eV energy (1 eV binding energy in ARPES data) in the experimental data is not found in the calculations performed using GGA, GGA + SOC, mBJ (not shown here) and mBJ + SOC methods. Moreover, the dispersion of the energy bands does not match with the ARPES results. These discrepancies may be related to the underestimation of the electron correlation effects in these methods.

**Figure 6:**
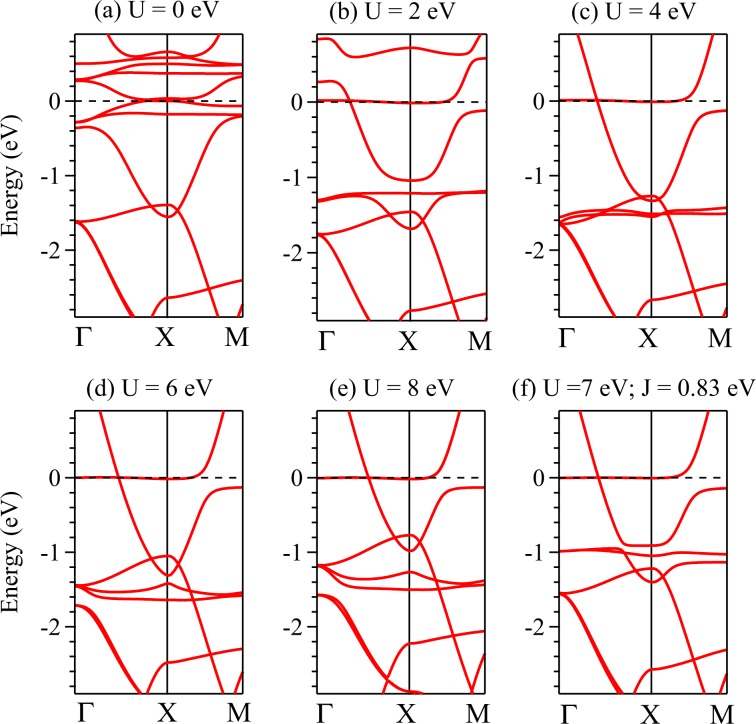
Energy band structure along Γ*XM* direction calculated using GGA + SOC + *U* method for (**a**) *U* = 0, (**b**) *U* = 2 eV, (**c**) *U* = 4 eV, (**d**) *U* = 6 eV, (**e**) *U* = 8 eV and *U* = 7 eV, *J* = 0.83 eV. The band structure in (**f**) correspond well with the ARPES results.

**Figure 7:**
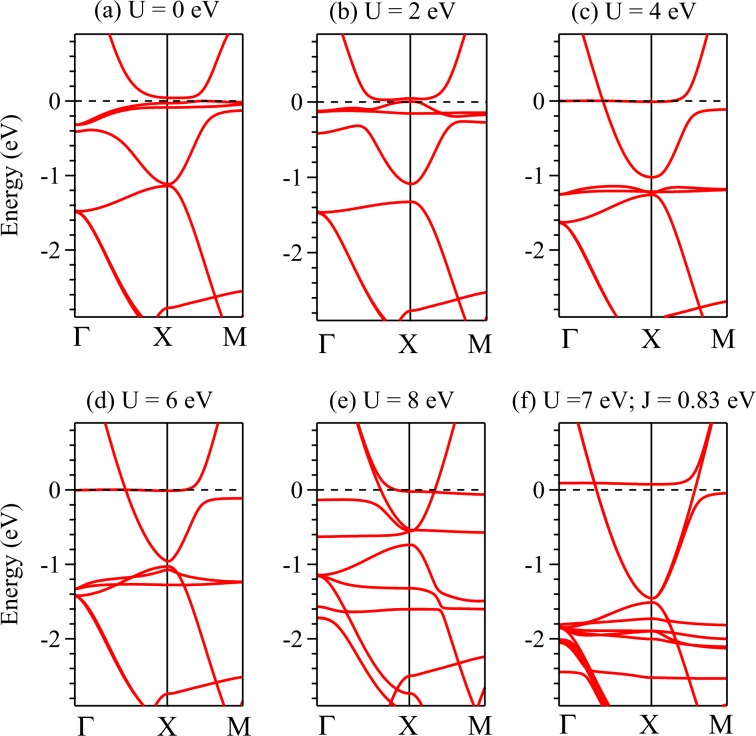
Energy band structure along Γ*XM* direction calculated using mBJ + SOC + *U* method for (**a**) *U* = 0, (**b**) *U* = 2 eV, (**c**) *U* = 4 eV, (**d**) *U* = 6 eV, (**e**) *U* = 8 eV and *U* = 7 eV, *J* = 0.83 eV. None of these cases could capture the scenario observed in ARPES results.

In order to investigate the role of electron correlation on the electronic structure within the density functional theory, we calculated the energy band structure following GGA + SOC + U and mBJ + SOC + U methods (*U* = electron-electron Coulomb repulsion strength). The results are shown in Figs. 6 and 7. We have focused on the *k*-vectors, Γ − *X* and *X* − *M* only, where the band cross the Fermi level and the experimental results are available in the literature^14,44,45^. We have performed the calculations for various values of *U* ranging from 2 eV to 10 eV for both GGA + SOC + *U* and mBJ + SOC + *U*.

In Fig. 6, we observe that with the increase in *U*, the 4*f*_5∕2_ bands gradually shift towards higher binding energies. There are significant other changes in the electronic structure due to incorporation of *U*. For example, most of the flat bands near Fermi level in the uncorrelated case shifts to higher energies with the increase in *U*. We observe signature of a flat 4*f* band in the proximity of the Fermi level for all values of *U* used in our calculations. From the calculations with various combinations of *U* and *J*, we find that the results for *U* = 7 eV and *J* = 0.83 eV provide the best description of the experimental results exhibiting signature of band inversion along *XM* direction and flat 4*f* bands at −1 eV. In addition, there is a flat band around −2.5 eV along *XM* direction.

The results from mBJ + SOC + *U* calculations appear significantly different from the results of GGA + SOC + *U* results and experimental data. For example, the data for *U* = 0 and 2 eV exhibit flat bands representing Sm 4*f* states near the Fermi level; the change in the band dispersion is found at different energy regimes. For *U* = 4 eV and 6 eV, the 4*f*_5∕2_ bands appear slightly below −1 eV energy. However, *U* = 8 eV exhibit flat bands spread over a large energy range leading to complex Sm 4*f* − B 2*p* hybridizations. The *k*-vector exhibiting band inversion scenario depends on the value of *U* considered in the calculations. While the energetics of the 4*f* bands for *U* = 4 eV seems closer to the experiments, the dispersive bands are significantly different from the experimental results. Overall, it was difficult to capture the band structure consistent with experiments using mBJ + SOC + *U* method.

It is to note here that the Becke-Roussel potential, ${v}_{x,\sigma }^{BR}({\bf{r}})$ was proposed to model the Coulomb potential due to an exchange hole^46^, which is very similar to the Slater potential. The mBJ potential was conceived by adding a semi-local correction term to the Becke-Roussel potential to capture the features like step structure and derivative discontinuity of the exchange correlation potential at integral particle number^47^ and is expressed as, ${v}_{x,\sigma }^{mBJ}({\bf{r}})=c{v}_{x,\sigma }^{BR}({\bf{r}})+(3c-2)\frac{1}{\pi }\sqrt{\frac{5}{12}}\sqrt{\frac{2{t}_{\sigma }({\bf{r}})}{{\rho }_{\sigma }({\bf{r}})}}$ where the electron density, ${\rho }_{\sigma }={\sum }_{i=1}^{{N}_{\sigma }}| {\psi }_{i,\sigma }{| }^{2}$, the kinetic-energy density, ${t}_{\sigma }=(1/2){\sum }_{i=1}^{{N}_{\sigma }}| \nabla {\psi }_{i,\sigma }{| }^{2}$, and $c=\alpha +\beta {(\frac{1}{{V}_{cell}}{\int }_{cell}\frac{| \nabla \rho ({{\bf{r}}}^{^{\prime} })| }{\rho ({{\bf{r}}}^{^{\prime} })}{d}^{3}{r}^{^{\prime} })}^{\frac{1}{2}}$. The values of *α* (= −0.012) and *β* (=1.023 (Bohr)^1∕2^) are fixed by comparing the theoretical results with the experimental data of a large number of materials. While this potential is quite successful in predicting the band gap of wide varieties of systems ranging from wide band gap insulators to correlated transition metal oxides^47^, polarizabilities in insulators with significant accuracy^48^, our results indicate that GGA calculations provide a better description of the energy band structure in the present case. In order to improve the theoretical representation of the electronic structure, one might require to tune the free parameters further; we hope that these results will provide incentive to initiate such studies in the future.

**Figure 8:**
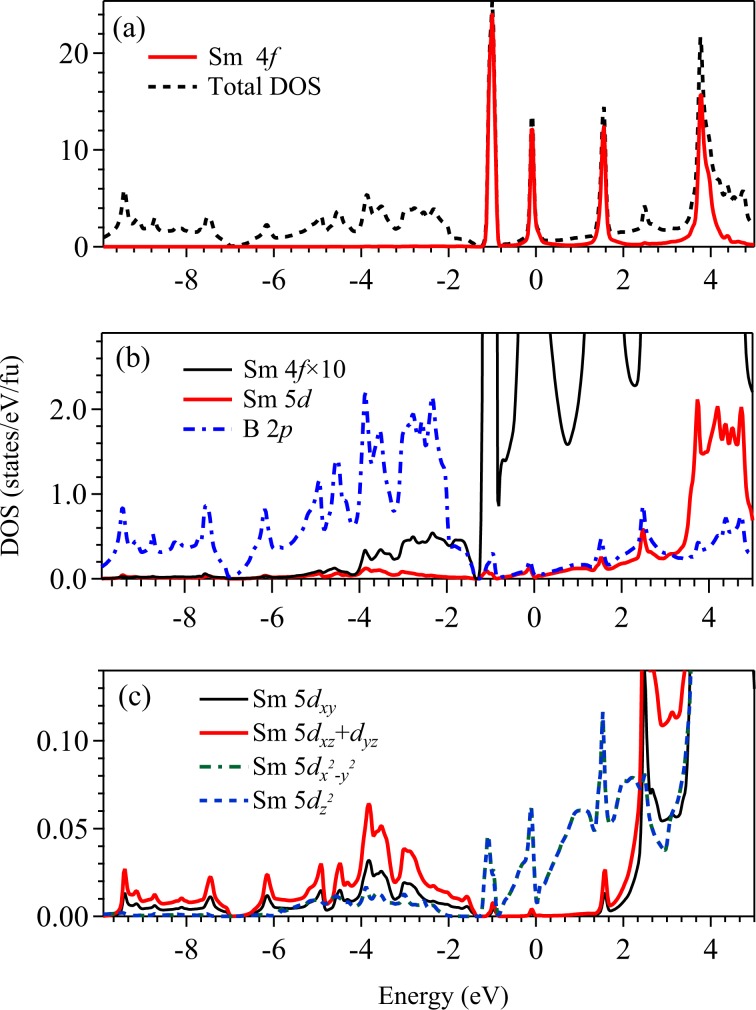
Calculated density of states using GGA + SOC + *U* method for *U* = 7 eV and *J* = 0.83 eV. (**a**) Total density of states (Total DOS) are shown by dashed line and the solid line represents the Sm 4*f* partial density of states (PDOS). (**b**) Sm 4*f* PDOS rescaled by 10 times is shown by thin black solid line. Sm 5*d* PDOS is shown by thick red solid line and B 2*p* PDOS is the blue dashed line. (**c**) PDOS of Sm 5*d*_*xy*_ (thin black solid line), (5*d*_*xz*_ + 5*d*_*yz*_) (thick red solid line), Sm 5${d}_{{x}^{2}-{y}^{2}}$ (green dot-dashed line) and Sm 5${d}_{{z}^{2}}$ (blue dashed line) are shown. Evidently, the contribution at the Fermi level is weak and contributed primarily by (5${d}_{{x}^{2}-{y}^{2}}$,5${d}_{{z}^{2}}$) states.

We now turn to the discussion of the detailed electronic structure for the case, which captures the experimental results well. The DOS obtained using GGA + SOC + *U* (*U* = 7 eV and *J* = 0.83 eV) is shown in Fig. 8. Sm 4*f*_7∕2_ features are observed to be shifted to higher energies, thus, rendering the region near the *E*_*F*_ dominated by Sm 4*f*_5∕2_ features. The DOS at the Fermi level is found to be finite as found in other hexaborides^49–51^ even after the application of GGA + SOC + *U*; there are significant Sm 4*f* contributions (97.3%) along with Sm 5*d* (0.46%) and B 2*p* contributions ( ~ 2%) at *E*_*F*_. There is a peak at around −1 eV, which is in good agreement with the *x*-ray photoemission spectra (XPS)^52^ and the ARPES spectra^14,44,45^.

In Fig. 8b, we show Sm 5*d*, B 2*p* and Sm 4*f* PDOS together where Sm 4*f* contribution is rescaled by 10 times for better comparison. It is clear that Sm 4*f* − B 2*p* mixing is stronger in this case compared to the results found in GGA calculations shown in Fig. 2. This is expected as the consideration of electron correlation brings the Sm 4*f* energies in closer proximity to B 2*p* energies. While Sm 5*d* states with *t*_2*g*_ symmetry hybridize strongly with the B 2*p* states, 5*d* states with *e*_*g*_ symmetry appear to mix with 4*f* states (see Fig. 8c).

**Figure 9:**
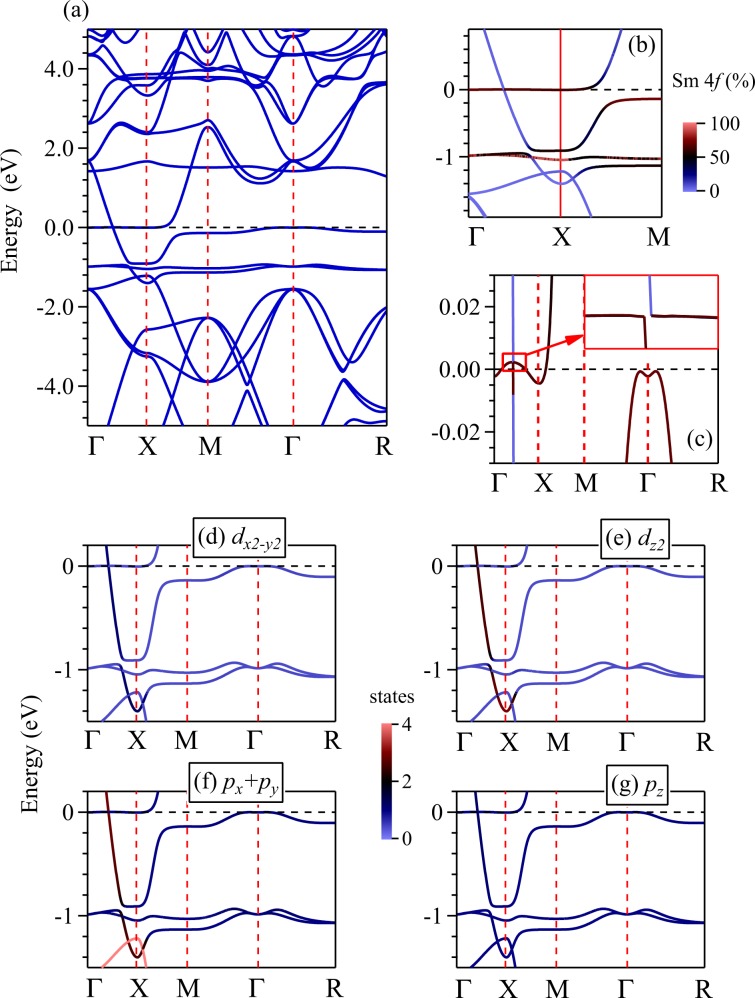
(**a**) Energy band structure calculated using GGA + SOC + *U* method for *U* = 7 eV, *J* = 0.83 eV. (**b**) Sm 4*f* character of the bands near the Fermi level are highlighted by the color plots exhibiting signature of band inversion. Weakly dispersive band at −1 eV arises primarily due to the Sm 4*f* states. (**c**) Near Fermi level region is shown in an expanded energy scale to reveal the band crossing. The color plot exhibit Sm 4*f* character of the bands crossing the Fermi level. Various 5*d*-symmetry of the energy bands obtained by projecting the eigenstates onto Sm 5*d* states are shown for (**d**) Sm 5${d}_{{x}^{2}-{y}^{2}}$, (**e**) Sm 5${d}_{{z}^{2}}$, (**f**) B (2*p*_*x*_ + 2*p*_*y*_) and (**g**) B 2*p*_*z*_ states.

The electronic band structure using GGA + SOC + *U* is shown in Fig. 9. The flat bands due to Sm 4*f*_5∕2_ states are observed near the Fermi level and near −1 eV energy. A highly dispersive band crosses the Fermi level along Γ*X*. A gap opens up at the Fermi level along *XM* direction due to the hybridization of Sm 4*f* states with the highly dispersive states having dominant B (2*p*_*x*_ + 2*p*_*y*_) character along with some Sm 5${d}_{{z}^{2}}$ character as evident from the plots in Fig. 9d–g. Based on DFT + *U* calculations using the VASP code, Chang *et al*. have shown that the hybridization gap in SmB_6_ between the localized Sm 4*f* bands and the conduction bands remains almost unchanged as *U* is varied up to values as large as 8 eV^41^. However, we observe significant influence of *U* and consideration of exchange correlation potential on the hybridization gap as can be anticipated in such a strongly correlated system. The band inversion like scenario near the Fermi level observed here is consistent with the conclusions from the experimental results.

The highly dispersive band crossing the Fermi level along Γ*X* line possesses dominant B (2*p*_*x*_ + 2*p*_*y*_) character and hybridizes with the Sm 4*f* band. The data in Fig. 9c exhibit an electron pocket primarily formed by the Sm 4*f* states around *X* point. A hole like bubble is observed along the Γ*X* line. No other band crossing is found in the data. The flat bands around −1 eV appear due to correlation induced effects among 4*f* electrons. The band crossing at *X*-point around −1.5 eV energy gets significantly modified due to strong enhancement of Sm 4*f* − B 2*p* hybridizations with the increase in *U*. Therefore, the bands shown in Fig. 9 possess strong mixed character consistent with the findings of mixed valency^53^.

**Figure 10:**
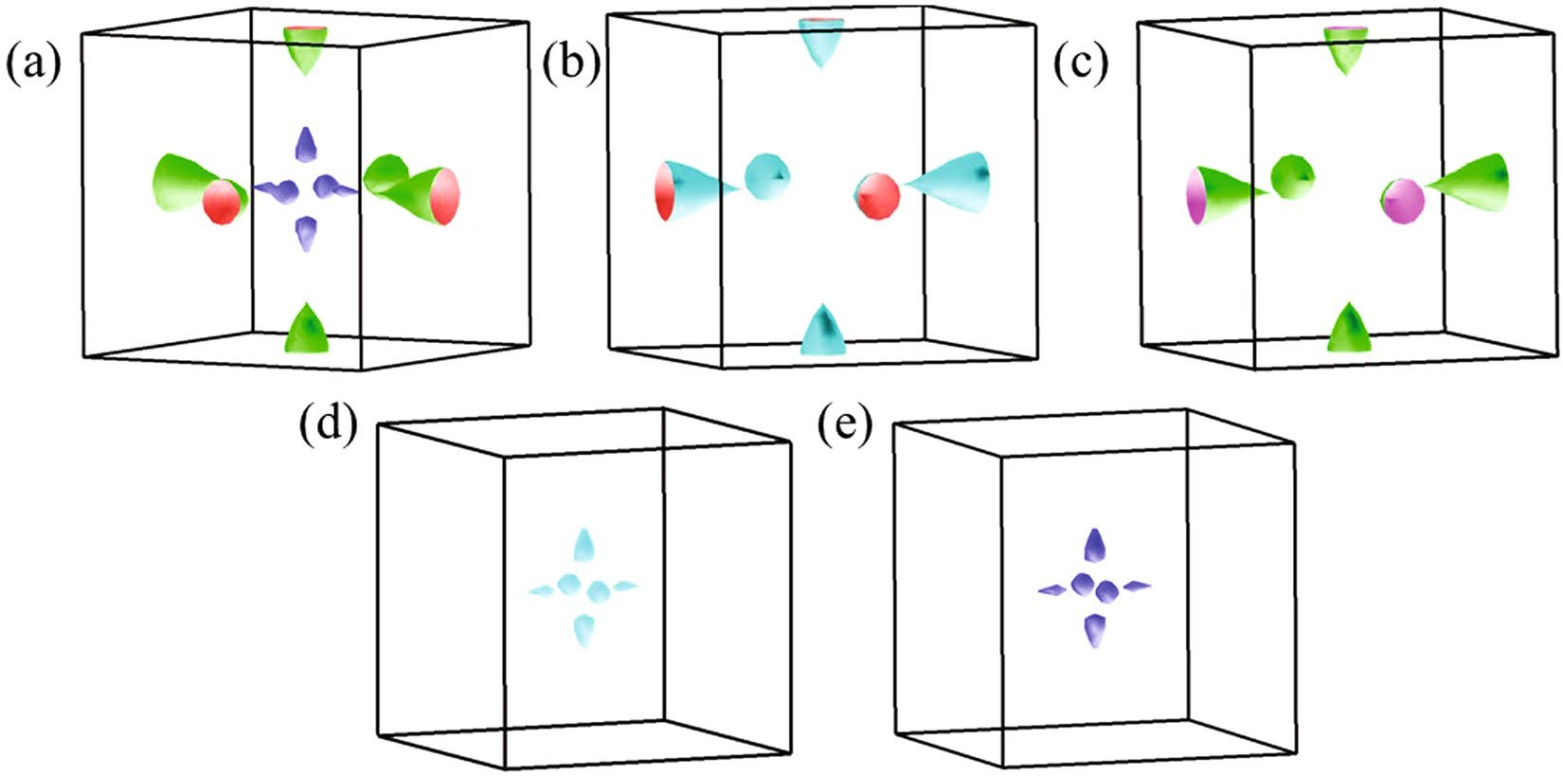
(**a**) Fermi surfaces derived using GGA + SOC + *U* method for *U* = 7 eV, *J* = 0.83 eV are shown. Electron pockets around *X* point are shown in (**b**) and (**c**). The hole pockets formed around Γ*X* vector are shown in (**d**) and (**e**). All these Fermi pockets are found to possess dominant 4*f* character.

The scenario discussed above in the band dispersion plots are well manifested in the Fermi surface shown in Fig. 10. The Fermi surface consists of four distinct Fermi sheets; two of them are due to the electron pockets formed around the *X* points and two are due to the hole pockets formed on the *X* − Γ line. These results are consistent with the high frequency quantum oscillations results^20,21^. It is to note here that the dynamical mean field theory (DMFT) often provides a good description of the electronic structure of correlated systems. Kim *et al*.^40^ have performed the band structure calculations of SmB_6_ using both DFT and DMFT methods, and concluded that the band structure obtained by using DMFT and DFT near the Fermi level are quite similar. Here, we find that B2*p*-Sm4*f* hybridization plays a key role in the electronic structure in this system and covalency can be captured well by DFT using suitable choice of interaction parameters and presumably the reason for remarkable representation of the experimental scenario^54,55^.

It is to note here that many experiments have shown deviation of the electronic properties of SmB_6_ from Kondo insulating scenario exhibiting it not to be a true insulator. Wakeham *et al*.^56^ have argued that for an insulator without any impurity states or magnetic excitations, the only contribution to the specific heat will be from phonons and so there will not be any linear temperature dependence to the specific heat. But the low temperature contribution to the specific heat is linear in temperature for SmB_6_^57,58^. Through measurement of the specific heat in the single crystals and a ground powder of SmB_6_, they showed that the residual linear term in the low temperature specific heat of SmB_6_ is predominantly a bulk property and does not originate from the conductive surface states^56^. In another experiment, Laurita *et al*. have used time domain terahertz spectroscopy to investigate the low energy optical conductivity within the hybridization gap of single crystals of SmB_6_ prepared from both optical floating zone and aluminium flux methods. They have also claimed that this material exhibits significant three dimensional bulk conduction band originating within the Kondo gap^59^. All these experimental results supports bulk metallicity in SmB_6_ as found in our study. Moreover, the finding of the Fermi surface in quantum oscillation measurements by Tan *et al*.^20^ and the ARPES results by Frantzeskakis *et al*.^16^ are consistent with our results providing confidence to the conclusions of our study. A recent work^60^ proposed a model requiring small electron doping and strong particle-hole asymmetry to explain the low temperature anomalies in SmB_6_. Our results show the presence of such states and particle-hole asymmetry in the electronic structure of pristine SmB_6_. In order to understand insulating transport, one should note that the Fermi level is pinned very close to the band edges with a tiny Fermi energy ( ≈ 4.5 meV). Thus, a small disorder can localize the electrons easily giving rise to an insulating behavior^61^.

## Conclusions

In Summary, we have performed detailed electronic structure calculations of SmB_6_ using different exchange correlation potentials, spin-orbit coupling and electron correlation strength. We find that Sm 4*f* multiplets calculated using GGA + SOC + *U* scheme provide a good description of the experimental ARPES data for the electron correlation strength of 7 eV and exchange interaction strength of 0.83 eV. The results reveal small electron pockets around *X*-point and hole pockets around Γ*X* line having dominant 4*f* character; the Fermi surfaces observed here are consistent with varied experimental results. In addition, we show that the B 2*p* contribution at *E*_*F*_ is about 4.5 times of the Sm 5*d* contributions. This suggests strong Sm 4*f*-B 2*p* hybridization in the electronic structure that is presumably responsible for the anomalous transport properties in this material. Evidently, electron correlation and spin-orbit coupling are indispensable besides exchange correlation potentials to derive the electronic properties of mixed valent Kondo insulators. These results are valuable for deeply understanding the exotic ground state properties arising from strong Coulomb repulsion among 4*f* electrons and their hybridization to conduction electrons.

## Methods

The electronic structure calculations were performed using the full-potential linearized augmented plane-wave (FLAPW) method as implemented in the WIEN2k software^62^. We have used the generalized-gradient approximation (GGA) for the exchange correlation functional proposed by Perdew-Burke-Ernzerhof (PBE)^63^, where the functional depends on local charge density as well as on the spatial variation of the charge density. In order to verify the sensitivity of the results on the choice of exchange correlation potentials, we have also calculated the electronic structure using modified Becke Johnson (mBJ) potential^47^, where the potential uses information from kinetic energy density in addition to the charge density. The mBJ potential corresponds to an orbital-independent semi-local exchange potential mimicking the orbital dependent behavior. It has been found to yield good description of the band gaps, effective masses and correct band ordering at time-reversal invariant momenta (TRIM), and are in good agreement with the improved many-body but more computation demanding GW (G = Green’s function and W = screened Coulomb interaction) calculations^64–66^. Calculations were performed with and without inclusion of electron correlation (*U* = electron-electron Coulomb repulsion strength) and spin-orbit coupling (SOC). From the experiments, it is observed that SmB_6_ does not show magnetic order down to 19 mK^67^. In order to simulate this, we have performed constrained magnetic calculations so that the magnetism of SmB_6_ is consistent with the experimental scenario. The Fermi surfaces were calculated using Xcrysden with 31 × 31 × 31 *k* mesh. The spin-orbit coupling was included self-consistently in the electronic structure calculations with a 17 × 17 × 17 *k*-mesh.
